# A Bayesian Framework for Functional Mapping through Joint Modeling of Longitudinal and Time-to-Event Data

**DOI:** 10.1155/2012/680634

**Published:** 2012-05-22

**Authors:** Kiranmoy Das, Runze Li, Zhongwen Huang, Junyi Gai, Rongling Wu

**Affiliations:** ^1^Department of Statistics, Temple University, Philadelphia, PA 19122, USA; ^2^Department of Statistics, The Pennsylvania State University, University Park, PA 16802, USA; ^3^Center for Statistical Genetics, The Pennsylvania State University, Hershey, PA 17033, USA; ^4^Department of Agronomy, Henan Institute of Science and Technology, Xinxiang 453003, China; ^5^National Center for Soybean Improvement, National Key Laboratory of Crop Genetics and Germplasm Enhancement, Soybean Research Institute, Nanjing Agricultural University, Nanjing 210095, China

## Abstract

The most powerful and comprehensive approach of study in modern biology is to understand the whole process of development and all events of importance to development which occur in the process. As a consequence, joint modeling of developmental processes and events has become one of the most demanding tasks in statistical research. Here, we propose a joint modeling framework for functional mapping of specific quantitative trait loci (QTLs) which controls developmental processes and the timing of development and their causal correlation over time. The joint model contains two submodels, one for a developmental process, known as a longitudinal trait, and the other for a developmental event, known as the time to event, which are connected through a QTL mapping framework. A nonparametric approach is used to model the mean and covariance function of the longitudinal trait while the traditional Cox proportional hazard (PH) model is used to model the event time. The joint model is applied to map QTLs that control whole-plant vegetative biomass growth and time to first flower in soybeans. Results show that this model should be broadly useful for detecting genes controlling physiological and pathological processes and other events of interest in biomedicine.

## 1. Introduction

To study biology, a classic approach is dimension reduction in which a biological phenomenon or process is dissected into several discrete features over time and space. Most efforts in the past decades have been made to understand biological details of individual features and then use knowledge from each feature to draw an inference about biology as a whole. There has been increasing recognition of the limitation of this approach because it fails to detect a rule that governs the transition from one feature to next, thus leading to a significant loss of information behind the development of a biological trait. More recently, tremendous developments in statistics and computer science have enabled scientists to model and compute the dynamic behavior of a biological phenomenon and construct a comprehensive view of how a cell, tissue, or organ grows and develops across the time-space scale. 

A statistical dynamic model, called functional mapping, is one of the products of such developments [1, 2]. The merit of functional mapping lies in its biological relevance to study the tempo-spatial pattern of change for the trait and further predict the physiological or pathological status of trait phenotype. Functional mapping has proven to be powerful for elucidating the dynamic genetic architecture of complex phenotypic traits by identifying when specific genes (known as quantitative trait loci or QTLs) involved turn on and turn off and how long they are expressed in a time course. With the advent of new automatic techniques that collect dynamic data in a cost-effective way, functional mapping can be anticipated to play an increasingly important role in shedding light on the genetic control mechanisms of complex traits or diseases. 

The statistical foundation of functional mapping is longitudinal data analysis or functional data analysis. There has been a considerable body of literature on statistical modeling of time-varying mean and covariance structure using various parametric, nonparametric, and semiparametric methods [3–7]. A joint mean-covariance model was proposed by Pourahmadi [8, 9], which shows some advantages over modeling the mean and covariance separately. Since the publication of the pioneering work by Laird and Ware [10], random effects model have been extensively used for longitudinal data analysis [11]. All these statistical approaches have been incorporated into functional mapping [12, 13], aiming to provide the most parsimonious estimates of QTL effects for a given data set. A Bayesian algorithm for functional mapping has been proposed recently by Liu and Wu [14].

The complexity of biology lies in the fact that no biological trait is isolated, rather every trait is affected by other traits through genes and environmental factors. For example, when a plant grows into a particular stage, reproductive behavior, such as flowering, starts to emerge as one of the important events in plant development. The time to first flower is highly associated with the amount of vegetative growth, depending on the environment where the plant is grown. Likewise, the time to recurrence of prostate cancer in humans is related with dynamic changes of prostate specific antigen level. How to jointly model longitudinal and time-to-event data within functional mapping has become an important issue for studying the common genetic basis of these processes and predicting events based on longitudinal traits.

Simultaneous modeling of longitudinal traits and time to events has been an active area in biostatistics during the past twenty years. A linear random effects model and EM estimation approach are proposed by Henderson et al. [15] for joint modeling. Guo and Carlin [16] made a comparative study between separate models and a joint model, showing that a joint model is more powerful when there is a strong correlation between the trait and the event. Wang and Taylor [17] developed a Bayesian method and MCMC algorithm for joint modeling of longitudinal and event time data and applied their algorithm on AIDS data. A review article by Tsiatis and Davidian [18] nicely summarizes the recent developments for such joint modeling.

By simply estimating the correlation between longitudinal traits and event time, Lin and Wu [19] developed a first model that connects these two aspects within functional mapping. However, they developed a likelihood-based framework where the covariance structure for the longitudinal trait was modelled by the known AR(1), and model parameters were estimated using maximum likelihood estimation. Taking advantage of event models, such as semiparametric Cox proportional hazard model, Weibull model, accelerated failure time (AFT) model, we here propose a sophisticated model for joint modeling of longitudinal trait and time to event to locate the QTLs which control the event via a dynamic trait. The detection of those QTLs that are common to these types of traits may help to prevent or accelerate the outcome by genetic approaches. Our model is constructed with a Bayesian paradigm and model parameters are estimated by the MCMC algorithm. Local polynomials are used to model the mean trajectory and generalized-linear-model- (GLM-) based approach is used to model the covariance matrix. The model is validated using a real example in which whole-plant biomass as a longitudinal trait measured at a series of discrete time points and the time to first flower as a time-to-event are jointly modeled through functional mapping. The statistical properties of the model applied to estimate QTL temporal effects in this example and its practical usefulness are investigated by simulation studies.

## 2. Joint Modeling Framework

### 2.1. Model for the Longitudinal Trait

Genetic mapping should be based on a segregating population, such as the backcross, F_2_, or recombinant inbred lines (RILS), initiated with two inbred lines each carrying an alternative allele. An RIL population is generated by self-crossing the hybrids of the two inbred lines continuously for 7-8 successive generations, which leads to two homozygous genotypes for alternative alleles at each locus. Methods for other designs can be derived similarly. Suppose a backcross has *n* progeny which is genotyped to construct a linkage map, aiming at locating putative QTLs that trigger significant effects on a longitudinal trait and its associated event. For each progeny, the trait is measured repeatedly at *T* different time points and a time-to-event is also recorded. At a specific time point *t*, the phenotypic value of the trait for progeny *i* affected by a putative QTL can be expressed by a linear model as follows:

(1) $$\begin{array}{c} y_{i}\left(t\right)=\overset{2}{\underset{j=1}{\sum }}x_{ij}u_{j}\left(t\right)+r_{i}\left(t\right)+e_{i}\left(t\right), \end{array}$$

where *x*_*ij*_ is an indicator variable for a possible QTL genotype of progeny *i* and defined as 1 if a particular QTL genotype *j* is indicated and 0 otherwise (*j* = 1 for QTL genotype *QQ* and 2 for genotype *qq*), *u*_*j*_(*t*) is the mean phenotypic value of QTL genotype *j* for progeny *i* at time *t*, *r*_*i*_(*t*) is the subject specific random effect, and *e*_*i*_(*t*) is the residual error assumed to follow a normal distribution with mean zero and covariance matrix *𝚺*. 

The central theme of functional mapping is to model the mean and covariance structures for the longitudinal trait efficiently. Here, we model the mean vector by polynomial function and the covariance matrix by an approach that guarantees the positive definiteness of the estimated covariance matrix. Without loss of generality, assume the response vector for progeny *i*, **y**_*i*_ = (*y*_*i*_(1),…, *y*_*i*_(*T*)), has mean 0 and covariance matrix *𝚺*. The response at time *t*, *y*_*i*_(*t*), can be predicted by its predecessors as the follows:

(2) $$\begin{array}{c} y_{i}\left(t\right)=\overset{t-1}{\underset{t^{\prime }=1}{\sum }}\phi_{t,t^{\prime }}y_{i}\left(t^{\prime }\right)+\epsilon_{i}\left(t\right), \end{array}$$

where *ϕ*_*t*,*t*′_ is the corresponding regression coefficient, *ϵ*_*i*_(*t*) is the prediction error for progeny *i* with mean = 0, and *σ* ^2^(*t*) is its variance. Assuming that *ϵ*_*i*_(*t*)'s are uncorrelated (Pourahmadi [8]), we get cov⁡(**ϵ**_*i*_) = *D*, a diagonal matrix with *σ* ^2^(*t*) being the *t*th diagonal element, where **ϵ**_*i*_ = (*ϵ*_*i*_(1),…, *ϵ*_*i*_(*T*))′ is the vector of prediction errors. Hence, the matrix representation of the above autoregression becomes

(3) $$\epsilon_{i}=My_{i},$$

where **M** is a lower triangular matrix with 1's in diagonal elements and −*ϕ*_*t*,*t*′_ in the (*t*, *t*′)th position. The above equation simply gives

(4) $$\begin{array}{c} \mathrm{cov}\left(\epsilon_{i}\right)=M\mathrm{cov}\left(y_{i}\right)M^{T}=M\text{Σ}M^{T}=D, \end{array}$$

which is related to the modified Cholesky decomposition of Σ [20].

Equation (4) will be considered as the basis for modeling the covariance structure, since this guarantees the estimated covariance matrix to be positive definite. Following Pourahmadi [8, 9], we model the mean vector, unconstrained variance parameters log⁡⁡*σ* ^2^(*t*), and dependence parameter *ϕ*_*t*,*t*′_, using a polynomial function of a particular order, expressed as

(5) $$\begin{array}{cc} & u_{j}\left(t\right)=\beta_{j0}+\beta_{j1}t+\beta_{j2}t^{2}+\cdots +\beta_{jr}t^{r}, \end{array}$$

(6) $$\begin{array}{cc} & r_{i}\left(t\right)=\theta_{i0}+\theta_{i1}t+\theta_{i2}t^{2}+\cdots +\theta_{im}t^{m}, \end{array}$$

(7) $$\begin{array}{cc} & \log\sigma_{t}^{2}=\eta_{0}+\eta_{1}t+\eta_{2}t^{2}+\cdots +\eta_{g}t^{g}, \end{array}$$

(8) $$\begin{array}{cc} & \phi_{t,t^{\prime }}=\delta_{0}+\delta_{1}\left(t-t^{\prime }\right)+\delta_{2}{\left(t-t\prime \right)}^{2}+\cdots +\delta_{h}{\left(t-t^{\prime }\right)}^{h},\\ & \;\;\;\;\left(t^{\prime }=1,2,\ldots ,t-1\right). \end{array}$$

The optimal (*r*, *m*, *g*, *h*) is determined from the information criteria (AIC/BIC). We note that different genotypes are assumed to have the same covariance structure but different means. Note that the above method of modeling the covariance structure for a longitudinal response is more robust than the traditional first-order autoregressive (AR(1)) or compound symmetry (CS) structure since real data might not show a parametric dependence structure. We refer to the proposed approach as GLM-based approach to estimate the covariance matrix.

Denote **β**_*j*_ = (*β*_*j*0_, *β*_*j*1_,…, *β*_*jr*_) for QTL genotype *j* and ***θ***_*i*_ = (*θ*_*i*0_, *θ*_*i*1_,…, *θ*_*im*_) for subject *i*. Then, the conditional mean function for progeny *i* carrying QTL genotype *j* (*j* = 1 or 2) for the given subject specific random effect (***θ***_*i*_) can be expressed as

(9) $$\begin{array}{c} \mu_{ij}=X_{i}^{\left(r\right)}\beta_{j}^{T}+X_{i}^{\left(m\right)}\theta_{i}^{T}, \end{array}$$

where

(10) $$\begin{array}{c} X_{i}^{\left(r\right)}=\left[\begin{array}{ccccc} 1 & t_{i1} & t_{i1}^{2} & \cdots & t_{i1}^{r}\\ 1 & t_{i2} & t_{i2}^{2} & \cdots & t_{i2}^{r}\\ \vdots & \vdots & \vdots & \ddots & \vdots \\ 1 & t_{i\tau } & t_{i\tau }^{2} & \cdots & t_{i\tau }^{r}\\ \vdots & \vdots & \vdots & \ddots & \vdots \\ 1 & t_{iT} & t_{iT}^{2} & \cdots & t_{iT}^{r} \end{array}\right] \end{array}.$$

Assume the vectors of subject specific random effects ***θ***_*i*_ follow *m*-variate normal distribution with mean 0 and covariance matrix *σ* ^2^ *I*_*m*_ and they are independent of the residual errors. Note that under this assumption, **y**_*i*_ | ***θ***_*i*_ will follow MVN(**X**_*i*_^(*r*)^ **β**_*j*_^*T*^ + **X**_*i*_^(*m*)^ ***θ***_*i*_^*T*^, Σ), and the marginal distribution of **y**_*i*_ will be MVN(**X**_*i*_^(*r*)^ **β**_*j*_^*T*^, Σ + *σ* ^2^ **X**_*i*_^(*m*)^ **X**_*i*_^(*m*)*T*^).

### 2.2. Model for the Event Time

We use *s*_*i*_ to denote the event time of progeny *i*. Since in the current situation, the event time is recorded for all progeny; no progeny is censored. Assuming a Cox proportional hazard model for this event, we get for progeny *i*,

(11) $$\lambda_{i}\left(t\right)=\lambda_{0}\left(t\right)\exp\left(\gamma \mu_{ij}\left(t\right)\right),$$

where *λ*_0_(*t*) denotes the baseline hazard at time *t*, *μ*_*ij*_(*t*) is the mean longitudinal trait at time *t* for given ***θ***_*i*_ when progeny *i* is of QTL genotype *j* and the regression coefficient *γ* represents the effect of the trait on the event time. The survival function for progeny *i* can be expressed in terms of the hazard function as *s*_*i*_(*t*) = exp⁡  [−∫_0_^*t*^ *λ*_*i*_(*u*)*du*].

The longitudinal model described above is linked to the hazard model by *γ*. If *γ* = 0, then the event is independent of the trait, and hence we should better fit separate models for the trait and the event. However, when *γ* is different from zero, a joint model performs better than the separate models [17]. For simplicity, the baseline hazard is assumed to be a step function, *λ*_0_(*t*) = *λ*_0*k*_ over a partition of the observed time scale [0, max(*s*_*i*_)] into *K* (possibly evenly spaced) intervals, (*t*_0_^*λ*^ = 0, *t*_1_^*λ*^, *t*_2_^*λ*^,…, *t*_*K*_^*λ*^). The value of *K* is usually not too large, possibly smaller than 10.

### 2.3. Likelihood for the Joint Model

Since the QTL genotype of a progeny is unknown, we use a mixture model to describe the likelihood of the progeny in terms of its possible underlying QTL genotypes [21]. The joint likelihood of unknown parameters Θ given the longitudinal trait **y** = (**y**_*i*_)_*i*=1_^*n*^ and event time **s** = (**s**_*i*_)_*i*=1_^*n*^ for all *n* progeny can be expressed as

(12) $$\begin{array}{cc} L\left(\Theta \;|\;y,s\right) & =\overset{n}{\underset{i=1}{\prod }}\left(\overset{2}{\underset{j=1}{\sum }}\omega_{j|i}\left[\pi \left(y_{i},s_{i}\;|\;Q_{i}=j,\theta_{i}\right)\right]\right)\\ & =\overset{n}{\underset{i=1}{\prod }}(\overset{2}{\underset{j=1}{\sum }}\omega_{j|i}\{\left[f\left(y_{i}\;|\;Q_{i}=j,\theta_{i}\right)\right]\\ & \;\;\;\;\;\;\times \left[\lambda_{i}\left(s_{i}\right)\exp\left(-\overset{s_{i}}{\underset{0}{\int }}\lambda_{i}\left(u\right)du\right)\right]\}), \end{array}$$

where *π*(·) denotes the joint density of the longitudinal trait and event time; *f*(.) denotes a multivariate normal with QTL genotype-specific mean ***μ***_*ij*_ modeled as (9) and covariance matrix *𝚺*; hazard function *λ*_*i*_(*s*_*i*_) is modeled as (11); and *ω*_*j*|*i*_ denotes the conditional probability of QTL genotype *j* given that the marker information of projeny *i* and *Q*_*i*_ is the QTL genotype for the *i*-th subject.

The QTL genotype is inferred from marker genotypes of the linkage map. Let *M*_*i*_ = (*M*_*i*1_,…, *M*_*im*_) be the *m*-marker genotypes for progeny *i*, D* the position of the putative QTL measured by its distance from the very first marker of an ordered linkage group, and *D*_*k*_ the distances between marker 1 and *k*. Assume that the QTL is located between marker *k* and *k* + 1. Then, the conditional probability of QTL genotype *j* given the genotype of these two markers that flank the QTL is expressed as

(13) $$\begin{array}{c} \omega_{j\;|\;i}=\text{Prob}\left(Q_{i}=j\;|\;D^{*},M_{ik},M_{i(k+1)},D_{k},D_{k+1}\right) \end{array}.$$

Note that, given the QTL locations D*, *D*_*k*_, and *D*_*k*+1_, one can compute *d*_1_, the distance of the QTL from marker *k* and *d*_2_, the distance of the QTL from marker *k* + 1 [22]. Using the Haldane map function, one can compute recombination fractions between marker *k* and QTL (*r*_1_), between QTL and marker *k* + 1 (*r*_2_), and between markers *k* and *k* + 1 (*r*) as follows:

(14) $$\begin{array}{c} r_{1}=\frac{1}{2}\left(1-e^{-2d_{1}}\right),\;\;r_{2}=\frac{1}{2}\left(1-e^{-2d_{2}}\right),\\ r=\frac{1}{2}\left(1-e^{-2d}\right), \end{array}$$

where *d* = *D*_*k*+1_ − *D*_*k*_ is the distance between marker *k* and *k* + 1. Wu et al. [22] provide a procedure for deriving the conditional probabilities of QTL genotypes given marker interval genotypes for the backcross, F_2_, and RIL populations, respectively.

Unknown parameters Θ in likelihood (12) contain QTL genotype-specific parameters **β**_*j*_, *σ* ^2^, the parameters that model the variance structure and dependence structure **η** = (*η*_0_, *η*_1_,…, *η*_*g*_) and **δ** = (*δ*_0_, *δ*_1_,…, *δ*_*h*_), as shown in models (7) and (8), respectively, the effect of the longitudinal trait on the event *γ*, QTL position D* and the baseline hazards *λ*_0*k*_.

### 2.4. Posterior Distribution and Sampling Procedure

We derive a Bayesian approach for estimating the unknown parameters. This will first need to specify the prior distributions for the parameters and, given the data and the priors, derive the posterior distribution over all the unknown parameters. For *β*_*j*_, we place a multivariate normal prior with zero mean and covariance matrix Σ_*β*_. An inverse gamma prior with parameters (*α*_1_,*α*_2_) is considered for *σ* ^2^. We consider a uniform prior on *γ* and uniform (0, *D*_*m*_) prior for the parameter D*. Independent Gamma (*a*, *b*) priors are taken for *λ*_0*k*_, for *k* = 1,…, *K*. Priors for **η** and **δ** are taken as MVN(0,Σ_*η*_) and MVN(0,Σ_*δ*_), respectively.

With the above priors and likelihood function, we have the joint posterior distribution for the parameters. In this case, it is quite straightforward to get the full conditional posterior distributions. Assume that the priors are independent for different parameters. Thus, we get the posterior density of *β*, *σ* ^2^, **γ**, D*, **η**, **δ**, ***λ***_0_ as

(15) $$\begin{array}{cc} & \pi \left(\beta ,\sigma^{2},\gamma ,D^{*},\eta ,\delta ,\lambda_{0}\;|\;y,s\right)\propto \pi \left(y,s\;|\;\beta ,\sigma^{2},\gamma ,D^{*},\eta ,\delta ,\lambda_{0}\right)\\ & \times \pi \left(\beta \right)\pi \left(\sigma^{2}\right)\pi \left(\gamma \right)\pi \left(D^{*}\right)\pi \left(\eta \right)\pi \left(\delta \right)\pi \left(\lambda_{0}\right). \end{array}$$

Assuming that priors for different genotypes are independent, we can express the above posterior distribution as

(16) $$\begin{array}{cc} & \pi \left(\beta ,\sigma^{2},\gamma ,D,\eta ,\delta ,\lambda_{0}\;|\;y,s\right)\\ & \propto \pi \left(y,s\;|\;\beta ,\sigma^{2},\gamma ,D^{*},\eta ,\delta ,\lambda_{0}\right)\\ & \times \left[\overset{2}{\underset{j=1}{\prod }}\pi \left(\beta_{j}\right)\right]\pi \left(\sigma^{2}\right)\pi \left(\gamma \right)\pi \left(D^{*}\right)\pi \left(\eta \right)\pi \left(\delta \right)\pi \left(\lambda_{0}\right). \end{array}$$

The full conditional distributions for the model parameters, as derived in the Appendix, are used to estimate the parameters using the MCMC algorithm. Note that the full conditional distribution for *β*_*j*_ is expressed as a product of a normal distribution term which comes from the longitudinal trait and two other terms from the hazard model. To update *β*_*j*_, therefore, we use a Metropolis-Hastings (MH) algorithm with a normal proposal density since it is a part of its posterior distribution. We also note that in the full conditionals of *η* and *δ*, normal distribution coming from the longitudinal part of the data is the main determinant. Hence for *η* and *δ*, we consider normal proposals with the current value of the parameter as the mean and covariance matrix as *𝚺*_*η*_ and *𝚺*_*δ*_, respectively. Selection of a good proposal density for *γ* is a bit tricky and we follow the recommendation given by Wang and Taylor [17]. By evaluating several choices for a good proposal, we consider a normal distribution with mean as the current state of the parameter and a suitable standard deviation in such a way that the proposed density gets well mixed with the target distribution (acceptance rate between 0.25 to 0.40). Because of conjugacy, we can directly simulate from the full conditional of *λ*_0*k*_'s.

The parameter D* which specifies the location of the QTL is updated following the idea of Satagopan et al. [23] by using the MH algorithm. A new value of D*, which we denote by *D* ^∗new^, is generated from Uniform $\left(\underset{}{\max}\left(0,D^{*}-\psi \right),\underset{}{\min}\left(D^{*}+\psi ,D_{m}\right)\right)$, where *ψ* is the tuning parameter. Denote this proposed distribution by *q*(D*, *D* ^∗new^). The proposed value will be considered as the new value of the chain with probability

(17) $$\begin{array}{cc} & \alpha \left(D^{*},D^{*\text{new}}\right)\\ & =\underset{}{\min}\left[1,\frac{\pi \left(D^{*\text{new}}\;|\;y,s,\beta ,\gamma ,\eta ,\delta ,\lambda_{0}\right)q\left(D^{*\text{new}},D^{*}\right)}{\pi \left(D^{*}\;|\;y,s,\beta ,\gamma ,\eta ,\delta ,\lambda_{0}\right)q\left(D^{*},D^{*\text{new}}\right)}\right]. \end{array}$$

We note that *π*(*D* ^∗new^ | …) ∝ *π*(*Q* | *D* ^∗new^, *M*)*π*(*D* ^∗new^) = ∏_*i*=1_^*n*^ *π*(*Q*_*i*_ | *D* ^∗new^, *M*_*i*_)*π*(*D* ^∗new^) and, similarly, *π*(D* | …) = ∏_*i*=1_^*n*^ *π*(*Q*_*i*_ | D*, *M*_*i*_)*π*(D*).

Because of the independence among *n* progeny, *Q* is updated by updating for each *Q*_*i*_ separately. For each progeny *i*, the full conditional density is in the form of a multinomial with the following cell probabilities:

(18) $$\begin{array}{c} \Pi \left(Q_{i}=j\;|\;y_{i},s_{i}\right)=\frac{\pi \left(Q_{i}=j\right)\pi \left(y_{i},s_{i}\;|\;Q_{i}=j\right)}{\sum_{j^{\prime }=1}^{2}\pi \left(Q_{i}=j^{\prime }\right)\pi \left(y_{i},s_{i}\;|\;Q_{i}=j^{\prime }\right)}. \end{array}$$

We can sample the QTL genotype directly from this full conditional density at each cycle. Details of the estimation procedure can be found in Satagopan et al. [23].

## 3. Application

### 3.1. Material and Analysis

The new model was applied to analyze a real data set for QTL mapping in soybeans. The mapping population contains 184 RILs derived from two cultivars, Nannong 1138-2 and Kefeng no. 1. A genetic linkage map of this population was first established by Zhang et al. [24] with 452 makers including RFLP, SSR, EST distributed among the 21 linkage group. This map was recently updated by adding some new SSR makers and dumping some unreliable markers. The new map contains 834 molecular makers covering a length of 2,308 cM in 24 linkage groups, with an average genetic distance of 2.85 cM between adjacent markers. Those markers with missing information were excluded from the analysis, leading to a total of 780 markers involved in our analysis.

**Figure 1:**
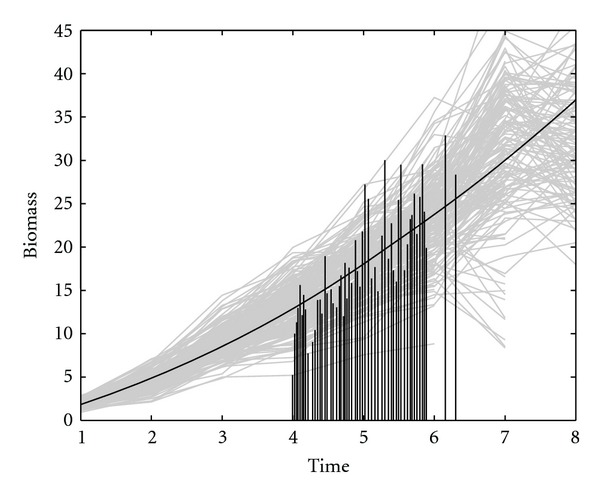
Whole-plant biomass growth trajectories for 184 soybean RILs. The time to first flower is indicated by a vertical line on each biomass growth curve. The black curve is the mean growth trajectory.

The plants and their parents were grown in a sample lattice design with two replicates at Jiangpu Soybean Experiment Station, Nanjing Agricultural University, China. After 20 days of seedling emergence, plant biomass (in gms.) were measured once every 5–10 days until most plants stopped height growth. A total of 8 measurements were taken for the biomass and the time to get the first flower in that growing season was also recorded for each plant.  Figure 1 shows the raw data, both the trait (biomass) and the event time for 184 plants.

Prior distributions for the model parameters were taken as follows. For genotype specific fixed effect *β*_*j*_, a multivariate normal prior was used with zero mean and a diagonal covariance matrix with all diagonal elements 100. For *σ* ^2^, we took *IG*(3,1) prior which has mean = 0.5 with small variance (0.25). A uniform prior *U*(−3.0, −0.1) was taken for *γ* following Wang and Taylor [17]. Observed time scale for the event time data was partitioned into 5 parts; that is, we took *K* = 5 and considered independent gamma (0.04, 1.0) priors for *λ*_01_,…, *λ*_05_, following Wang and Taylor [17]. Uniform prior was taken for D*. For *η* and *δ*, we considered multivariate normal priors with zero means and diagonal covariance matrices with diagonal elements 30 and 20, respectively. To investigate the effect of prior distributions on the estimation method, we did a sensitivity analysis. Considering different sets of priors, we fitted our model many times and it turned out the estimation is almost insensitive to the choice of priors. Hence the choice of our priors (even with huge variances for *β*, *η*, and *δ*) does not affect much the estimation of the model parameters.

We fitted our joint model as described in Section 2, by MCMC sampling. We ran chains 120,000 times. To remove the effect of the starting values, we excluded first 20,000 burn-in iterations. With the remaining 100,000 iterations, we estimated the posterior distributions and the parameters were estimated by the posterior mean and also calculated the sample standard deviations for the posterior densities. For the MH algorithm, the acceptance rates were 0.31, 0.26, 0.25, and 0.30 for *σ* ^2^, *γ*, *η*, and *δ*, respectively.

Since our model is complex, we perform several standard diagnostic tests to assess the convergence of the Markov chains. First, we use the method proposed by Brooks and Gelman [25]. Considering five different chains with different starting points and discarding the burn-in iterations, we computed multivariate potential scale reduction factors (MPSRF) to assess the convergence of the chains. Starting points for the model parameters were drawn from the respective priors. The computed values of this statistic get stabilized near 1 after 60,000 iterations for our model parameters, which indicates convergence of the chains.

Second, we perform Geweke test which compares the earlier part of the markov chain to the later part for assessing convergence. After deleting the burn-in iterations, from the remaining 100,000 iterations, we take out two subsequences; the first 50,000 and the last 50,000 iterations. Also consistent spectral density estimates at zero frequency are calculated to compute the *z*-scores. The calculated *P*values for our model parameters are above 0.18, which indicates a small absolute *z*-scores assessing the convergence of our chains. A detailed discussion of this method can be found in Geweke [26].

Finally, we perform the Heidelberger and Welch test as proposed by Heidelberger and Welch [27]. This test has two parts: a stationary test and a half-width test. Our chains after deletion of the burn-in iterations, pass the stationary test. To assess whether the number of iterations is adequate to estimate the parameters accurately, we calculate relative half-width (RHW). We consider the default *α* value (0.05) and predetermined tolerance value is taken as 0.1. For all our model parameters, the calculated RHW is in between 0.045 and 0.081. This indicates that we have enough iterations to estimate the model parameters with 95% confidence under tolerance of 0.1.

### 3.2. Results

**Figure 2:**
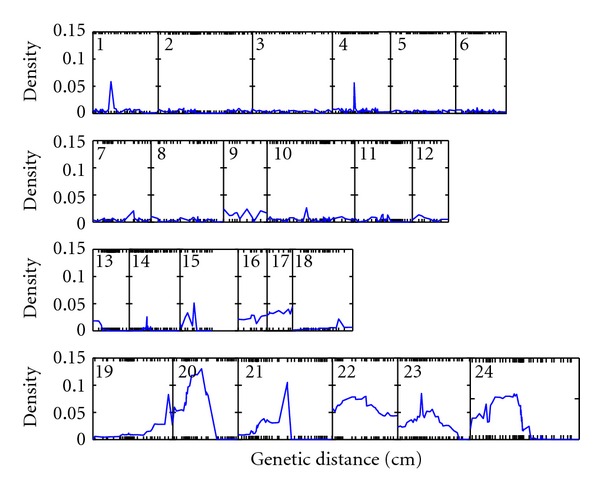
Marginal posterior plot for QTL locations over 24 linkage groups. Marker locations are indicated by ticks on the *x*-axis.

Figure 1 is the growth trajectories of whole-plant biomass over time for all RILs, in which the times to first flower for each RIL are also indicated. There are great variability found for these two traits among RILs. In the exploratory data analysis, we computed BIC values for different orders for the triplet (*r*, *m*, *g*, *h*), showing that the BIC value is smallest for the order (2,2, 2,2), that is, second order polynomials can best fit the the mean, variance, and dependence structures (Table 1). By scanning for the existence of QTLs over the genetic linkage map, we obtained the posterior distribution of the model parameters and estimate marginal posterior distributions of the QTL locations (D*) for all 24 linkage groups (Figure 2).

We observed posterior peaks in linkage groups 1, 4, 15, 19, 20, 21, and 23. To draw inference about the existence of a putative QTL in each of these groups, we computed the Bayes Factor (BF), defined as

(19) $$\begin{array}{c} \text{BF}=\frac{P\left(Y,S\;|\;\kappa =0\right)}{P\left(Y,S\;|\;\kappa =1\right)}, \end{array}$$

where *κ* denotes the number of QTLs in that particular group. Following Jeffrey's scale, the BFS with value smaller than 1 gives strong evidence against the null hypothesis and higher than 10 gives enough evidence for the null hypothesis. Note that in this case, we are testing the existence of no QTL (null) versus the existence of one QTL (alternative) for each linkage group. Here, no QTL in a group means *β*_1_ = *β*_2_, for that group. So, in order to compute BF we run our MCMC twice, first under the null and then under the alternative. The calculated BF for the above 7 linkage groups were 0.2781, 11.274, 13.493, 10.610, 0.5913, 11.475, and 0.7953, respectively, implying the existence of QTL in groups 1, 20, and 23.  Table 2 provides genotype-specific mean parameters for the QTLs located on linkage groups 1, 20, and 23, along with their 95 percent credible intervals (C.I.).

Since the nature of our model is complex and our estimation is based on MCMC, we perform posterior predictive check for the aforementioned 7 linkage groups. We simulate observations to get the posterior predictive distribution. Let Θ be the set of all model parameters and **D** ^rep^ = (**y** ^rep^, **s** ^rep^) be the replicated data. Then given the data **D** = (**y**, **s**), the posterior predictive distribution of **D** ^rep^ is given by *p*(**D** ^rep^ | **D**) = ∫*p*(**D** ^rep^ | Θ)*p*(Θ | **D**)*d*Θ.

One can simulate from the posterior predictive distribution using the following two steps. First from the posterior distributions of the model parameters, simulate *m* values (vectors) of Θ. Next for each value of Θ, simulate a value (vector) **D** ^rep^ from the likelihood. The *m* values (vectors) of **D** ^rep^ drawn in this way will essentially come from posterior predictive distribution *p*(**D** ^rep^ | **D**).

We simulate 100 draws (*m*=100) from the posterior predictive distribution and then apply proposed joint analysis and estimate the model parameters using MCMC as described earlier. We compute BF for each of those 7 linkage groups by considering the problem of testing the existence of no QTL (null) versus the existence of one QTL (alternative).  Table 3 shows the average BF (with estimated SE) for each linkage group and the existence of QTL in groups 1, 20, and 23 is quite evident.

Heritability (broad-sense) for the traits is estimated from the data, as the proportion of phenotypic variance attributable to genetic variance. The estimated heritability in our case is 32.6%. Also we compute the percentage of variance explained by three identified QTLs. It turns out the QTLs identified in linkage groups 1, 20, and 23 explain 6.8%, 14.3% and 11.4% of the total variance, respectively.

**Figure 3:**
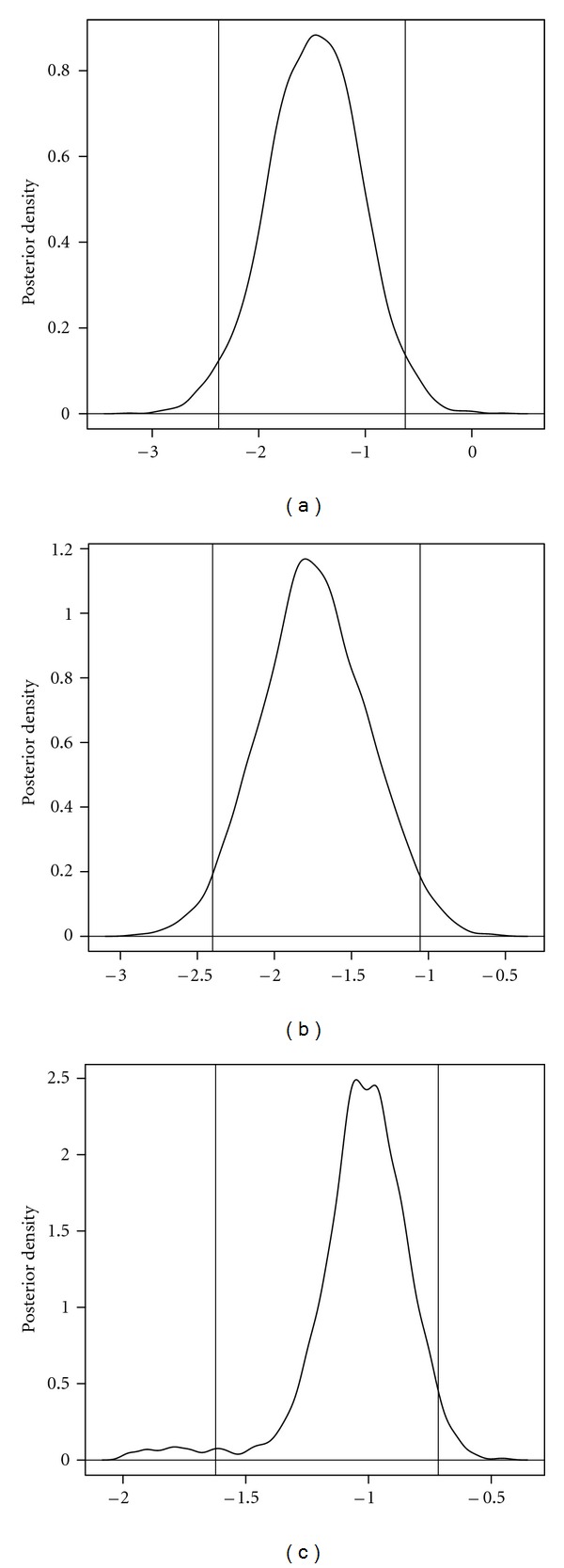
Marginal posterior plot for *γ* for linkage groups 1 (a), 20 (b), and 23 (c).

We show the marginal posterior plots with 95% credible intervals for the parameter *γ* in Figure 3. Note that for all three groups, the estimates and the confidence intervals are in the negative part which indicates a negative relationship between the trait and the event time. Biologically this is sensible since the plants with higher body mass will take less time to have the first flower compared to the plants with lower body masses.

**Figure 4:**
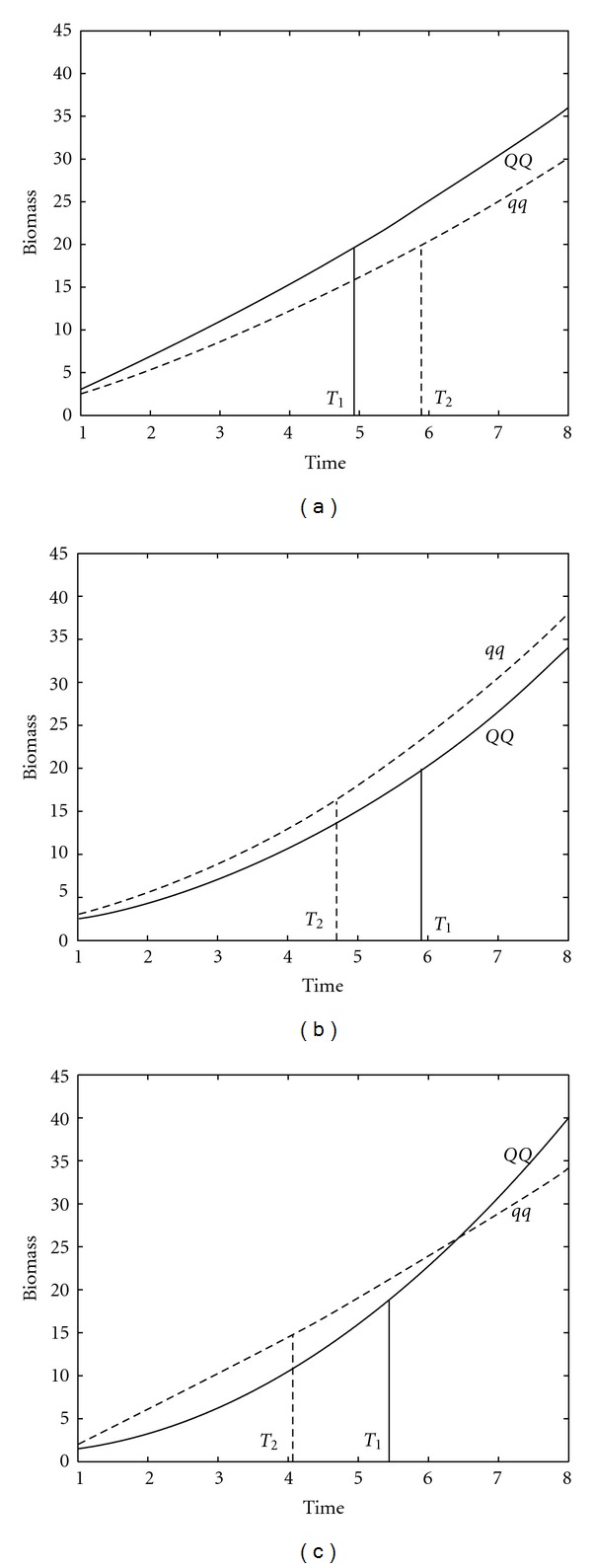
Whole-plant biomass growth trajectories and times to first flower (T_1_ and T_2_) for two different genotypes at each of the QTLs detected on linkage groups 1 (a), 20 (b), and 23 (c). Genotypes *QQ* inherit two alleles from parent Nannong 1138-2, whereas genotype *qq* inherits two alleles from parent Kefeng no. 1.

Figure 4 illustrates genotypic differences in whole-plant biomass trajectory and the time to first flower for the three QTLs detected. At the QTL on linkage group 1, the allele (*Q*) inherited from parent Nannong 1138-2 leads to increased biomass growth and earlier flowering than the allele (*q*) from parent Kefeng no. 1. The inverse pattern is observed for the QTL on linkage group 20. The QTL on linkage group 23 alters its direction of genetic effect. Affected by the first two QTLs, poor vegetative biomass growth in plants stimulates early flowering (Figures 4(a) and 4(b)). Yet, the QTL on linkage group 23 makes the fast-growing genotype to flower earlier than the slow-growing genotype (Figure 4(c)).

## 4. Simulation Study

We performed simulation studies to study the statistical properties of the joint model. We assumed an RIL design of 200 progeny and simulated 11 evenly spaced markers on a linkage group of length 100 cM. A QTL is located at 43 cM from the very first marker of the linkage group. To reflect a practical problem, we used parameter estimates of the soybean QTL detected in linkage group 20 as true values to simulate the data, allowing the covariance structure. Time-dependent phenotypic values were assumed to follow a multivariate normal distribution and the event times were taken the same as the soybean data. To make a comparison, we analyzed the simulated data using our nonparametric GLM-based covariance structure and the traditional AR(1) and CS covariance structures.

The prior distributions for the model parameters were taken in the same way as discussed in Section 3.1. A uniform prior on (0,100) was considered for D*. For each situation, we ran Markov chains 120,000 iterations and initial 20,000 burn-in iterations were discarded. Model parameters were estimated from the posterior distributions on the basis of remaining 100,000 iterations. The computed BF was 0.649 giving a strong evidence against the null hypothesis.  Table 4 shows the means of the Bayesian estimates of model parameters with their respective Monte Carlo standard errors, (MCSE). It can be seen that the estimates are quite close to the actual values with a reasonably small standard errors which justifies the accuracy and precision of our estimation procedure. However, our GLM-based approach provides better estimation of parameters than AR(1) and CS-based approaches.

**Figure 5:**
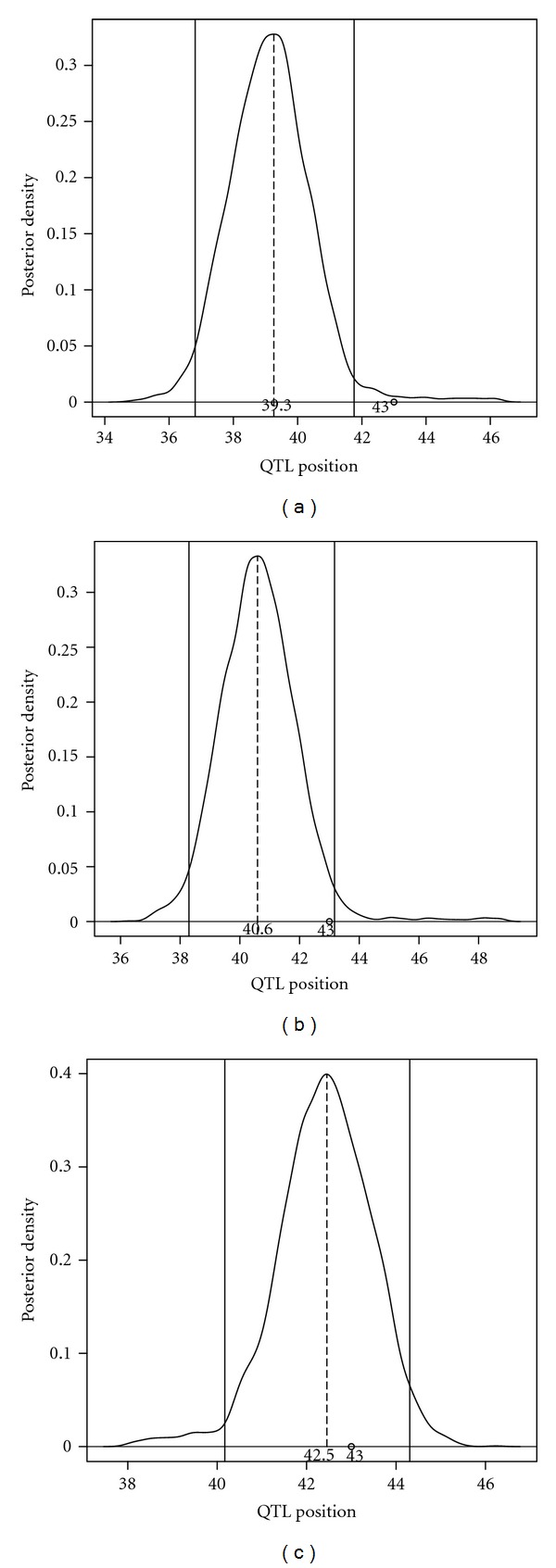
The Bayesian estimate of QTL location (indicated by dash vertical lines) from simulation studies under different covariance structures, AR(1) (a), CS (b), and nonparametric (c). The true QTL location is at 43 cM from the very first marker of the linkage group.

Figure 5 elucidates the marginal posterior plot for the QTL location under three different covariance structures. It is found that both AR(1)- and CS-based models provide the peaks at wrong locations, whereas GLM-based nonparametric covariance structure locates QTL more accurately in which case the length of the credible interval is narrower than those obtained from the former two structures. This provides numerical evidence that the proposed GLM-based model has better precision of QTL localization.

We perform further simulation studies to assess the reliability of BF in our data application. For each of the linkage groups 1, 20, and 23, we simulate data under the “null” model. As mentioned earlier, under the “null” model, *β*_1_ = *β*_2_. For group 1, we consider the null model *β*_1_ = *β*_2_ = (−0.4762, 3,3214, 0.1548), for group 20 it is *β*_1_ = *β*_2_ = (−1.1524, 2.6829, 0.2295), and for group 23, our null model is given as *β*_1_ = *β*_2_ = (0.4190, 0.5971, 0.5438). The computed BFS for these three groups are 34.55, 46.79, and 41.86, respectively, suggesting strong evidence for the null.

## 5. Discussion

 Tools to reveal the secret of life should reflect the dynamic nature of life. More recently, a series of statistical models have been developed to map quantitative trait loci (QTLs) that control the dynamic process of a complex trait [1, 2, 22, 28]. These so-called functional mapping models integrate mathematical aspects of biological processes into a statistical framework derived to map complex trait QTLs and have proved to be useful for detecting and identifying genes and genetic interactions involved in quantitative genetic variation for plant height, plant rooting ability, and animal body mass. Functional mapping is also flexible to incorporate complex biological phenomena, such as genotype-environment interactions and allometric scaling providing powerful means for addressing biological questions of fundamental importance.

In this paper, we develop a new version of functional mapping that can map QTLs for developmental events affected by organismic growth trajectories in time. This version is benefited from recent statistical developments for joint modeling of longitudinal traits and event time [16–18, 29]. In the presence of strong correlation between a longitudinal trait and event, a joint model performs better than submodels separately for a single trait. In the joint modeling framework for these two types of traits, we applied a GLM-based approach to model the covariance structure and local polynomials for the mean curves. Bayesian estimation method using the MCMC algorithm was used since it is computationally much simpler than a likelihood-based approach. Simulation results show the effectiveness of GLM-based covariance model compared to traditional parametric compound symmetry or autoregressive structure.

Our joint model, embedded within functional mapping, promotes the study of testing how QTLs pleiotropically affect different biological processes and how one trait is predicted by other traits through genetic information. The application of the new model to soybean mapping data does not only validate its usefulness and utilization, but also gains new insight into the genetic and developmental regulation of trait correlations in plants. There is no doubt that the new model can be modified to study the genetic associations between HIV dynamics and the time to death as well as prostate specific antigen change and the time to recurrence of prostate. However, there is much room for modifying this model. First, to clearly describe our idea, we assume one QTL at a time for trait control. Epistatic interactions between multiple QTLs may play an important role in trait development as well as in correlations between longitudinal traits and events. Second, from a dynamic systems perspective, we need to model dynamic correlations among multiple longitudinal traits and multiple events. Third, with the availability of efficient genotyping techniques, our model should accommodate a high-dimension model selection scheme to identify significant genetic variants from a flood of marker data.

## Figures and Tables

**Table 1:** BIC values for selecting the optimum (*r*, *m*, *g*, *h*) to model the mean-covariance structures for whole-plant biomass growth trajectories and the time to first flower in soybeans.

(*r*, *m*, *g*, *h*)	BIC
(3,3, 3,2)	7.18
(3,2, 3,2)	4.15
(3,3, 2,2)	4.26
(3,2, 2,2)	3.91
(2,2, 2,2)	**2.03**
(2,3, 2,2)	2.87
(2,2, 3,2)	3.01
(2,3, 3,2)	4.26
(3,3, 3,3)	4.66
(3,2, 3,3)	3.17
(3,3, 2,3)	3.93
(3,2, 2,3)	4.05
(2,2, 2,3)	3.34
(2,3, 2,3)	5.16
(2,2, 3,3)	4.88
(2,3, 3,3)	3.16

**Table 2:** Estimates of the parameters that describe genotype-specific biomass growth trajectories and QTL locations on linkage groups 1, 20, and 23, with 95% credible intervals.

Parameter	Group 1			Group 20			Group 23	
	estimate	C.I.		estimate	C.I.		estimate	C.I.
*β*_10_	−0.4762	(−0.5081, −0.4442)		−1.1524	(−1.1641, −1.1405)		0.4190	(0.3897, 0.4484)
*β*_11_	3.3214	(3.3023, 3.3404)		2.6829	(2.6463, 2.7194)		0.5971	(0.5566, 0.6377)
*β*_12_	0.1548	(0.1363, 0.1731)		0.2295	(0.2020, 0.2570)		0.5438	(0.5176, 0.5701)
*β*_20_	−5.4762	(−5.4788, −5.4734)		−3.1004	(−3.0231, −2.9768)		−2.3571	(−2.3701, −2.3442)
*β*_21_	8.4464	(8.4381, 8.4546)		5.3250	(5.3159, 5.3340)		4.4660	(4.4294, 4.5028)
*β*_22_	−0.4702	(−0.5011, −0.4392)		−0.0250	(−0.0618, 0.0118)		0.0410	(0.0390, 0.0432)
Marker								
Interval	Sat-356–B30T			GNE097b–A199H			LC4-4T–Sat-280	
D*	30.810	(29.1934, 31.7150)		49.600	(48.7515, 50.0726)		39.472	(38.8143, 40.1863)

**Table 3:** Posterior predictive check for 7 linkage groups.

Linkage group	BF (from actual data)	BF with SE (from posterior predictive distribution)
1	0.2781	0.2659 (0.15)
4	11.274	12.086 (1.76)
15	13.493	12.962 (2.17)
19	10.610	11.138 (1.56)
20	0.5913	0.6281 (0.73)
21	11.475	12.183 (1.18)
23	0.7953	0.7682 (0.89)

**Table 4:** Simulation results for genotypic-mean parameters and QTL locations under different covariance structures, AR(1)-, CS- and GLM-based approach.

Parameter		AR(1)			CS			GLM-based approach	
	Actual value	Estimate	MCSE		Estimate	MCSE		Estimate	MCSE
*β*_10_	−1.1524	−1.1872	0.0871		−1.0982	0.1094		−1.1667	0.0361
*β*_11_	2.6829	2.5391	0.0502		2.6103	0.0495		2.7001	0.0103
*β*_12_	0.2295	0.2288	0.0805		0.2301	0.0302		0.2291	0.0113
*β*_20_	−3.1004	−3.1204	0.0307		−3.093	0.1025		−3.1255	0.1011
*β*_21_	5.3250	5.3140	0.0291		5.2998	0.1130		5.3433	0.0405
*β*_22_	−0.0250	−0.0241	0.1302		−0.0257	0.1035		−0.0244	0.0603
D*	43.00	39.32	2.3561		40.61	1.5694		42.48	1.1572

## Appendix

Denote *β*_−*j*_ = (*β*_*j*′_: *j*′ = 1,2; *j*′ ≠ *j*), *S*(*t*) = exp⁡(−∫_0_^*t*^ *λ*(*u*)*du*), *h*(*t*) = (1, *t*, *t* ^2^,…, *t* ^*r*^), and *g*(*t*) = (1, *t*, *t* ^2^,…, *t* ^*m*^). Let *m*_*j*_ be the number of progeny that carries QTL genotype *j*. We derive the full conditional distributions for the unknown parameters as

(A.1) $$\begin{array}{cc} & \pi \left(\beta_{j}\;|\;y,s,\sigma^{2},\gamma ,D,\eta ,\delta ,\lambda_{0},\beta_{-j}\right)\\ & \propto \pi \left(\beta_{j}\right)\pi \left(y,s\;|\;\beta ,\sigma^{2},\gamma ,D,\eta ,\delta ,\lambda_{0}\right)\\ & \propto \pi \left(\beta_{j}\right)\pi \left(s\;|\;y,\beta ,\sigma^{2},\gamma ,D,\eta ,\delta ,\lambda_{0}\right)\\ & \times \pi \left(y\;|\;\beta ,\sigma^{2},\gamma ,D,\eta ,\delta ,\lambda_{0}\right)\\ & \propto \exp[-\frac{1}{2}\beta_{j}{\text{Σ}}_{\beta }^{-1}\beta_{j}^{T}-\frac{1}{2}\overset{m_{j}}{\underset{i=1}{\sum }}{\left(y_{i}-X_{i}^{\left(r\right)}\beta_{j}^{T}\right)}^{T}\\ & \;\;\;\;{\times \text{Σ}}^{-1}\left(y_{i}-X_{i}^{\left(r\right)}\beta_{j}^{T}\right)]\times \left[\exp\left(\gamma \overset{m_{j}}{\underset{i=1}{\sum }}h_{i}\left(s_{i}\right)\beta_{j}^{T}\right)\right]\\ & \;\times \left[\overset{m_{j}}{\underset{i=1}{\prod }}S_{i}\left(s_{i}\right)\right]\\ & =\exp[-\frac{1}{2}\beta_{j}{\text{Σ}}_{\beta }^{-1}\beta_{j}^{T}-\frac{1}{2}\overset{m_{j}}{\underset{i=1}{\sum }}{\left(y_{i}-X_{i}^{\left(r\right)}\beta_{j}^{T}\right)}^{T}\\ & \;\;\;\;\times {\text{Σ}}^{-1}\left(y_{i}-X_{i}^{\left(r\right)}\beta_{j}^{T}\right)]\times \left[\exp\left(\gamma \overset{m_{j}}{\underset{i=1}{\sum }}h_{i}\left(s_{i}\right)\beta_{j}^{T}\right)\right]\\ & \;\times \left[\overset{m_{j}}{\underset{i=1}{\prod }}S_{i}\left(s_{i}\right)\right]\\ & \propto \exp[\beta_{j}{\text{Σ}}_{\beta }^{-1}\beta_{j}^{T}+\overset{m_{j}}{\underset{i=1}{\sum }}\left(\beta_{j}X_{i}^{(r)T}{\text{Σ}}^{-1}X_{i}^{\left(r\right)}\beta_{j}^{T}\right)\\ & \;\;\;-2\overset{m_{j}}{\underset{i=1}{\sum }}\left(y_{i}^{T}{\text{Σ}}^{-1}X_{i}^{\left(r\right)}\beta_{j}^{T}\right)]\left[\exp\left(\gamma \overset{m_{j}}{\underset{i=1}{\sum }}h_{i}\left(s_{i}\right)\beta_{j}^{T}\right)\right]\\ & \;\;\times \left[\overset{m_{j}}{\underset{i=1}{\prod }}S_{i}\left(s_{i}\right)\right]\\ & =\exp[\beta_{j}\left(\Sigma_{\beta }^{-1}+\overset{m_{j}}{\underset{i=1}{\sum }}X_{i}^{(r)T}{\text{Σ}}^{-1}X_{i}^{\left(r\right)}\right)\beta_{j}^{T}-2\overset{m_{j}}{\underset{i=1}{\sum }}\left(y_{i}^{T}{\text{Σ}}^{-1}X_{i}^{\left(r\right)}\beta_{j}^{T}\right)]\\ & \;\times \left[\exp\left(\gamma \overset{m_{j}}{\underset{i=1}{\sum }}h_{i}\left(s_{i}\right)\beta_{j}^{T}\right)\right]\left[\overset{m_{j}}{\underset{i=1}{\prod }}S_{i}\left(s_{i}\right)\right]. \end{array}$$

Hence, we have

(A.2) $$\begin{array}{cc} & \pi \left(\beta_{j}\;|\;.\right):\text{MVN}\left[\beta_{j},M_{\beta },V_{\beta }\right]\\ & \;\;\times \left[\exp\left(\gamma \overset{m_{j}}{\underset{i=1}{\sum }}h_{i}\left(s_{i}\right)\beta_{j}^{T}\right)\right]\left[\overset{m_{j}}{\underset{i=1}{\prod }}S_{i}\left(s_{i}\right)\right], \end{array}$$

where

(A.3) $$\begin{array}{cc} & M_{\beta }=\left[{\left({\text{Σ}}_{\beta }^{-1}+\overset{m_{j}}{\underset{i=1}{\sum }}X_{i}^{(r)T}{\text{Σ}}^{-1}X_{i}^{\left(r\right)}\right)}^{-1}\left(\overset{m_{j}}{\underset{i=1}{\sum }}X_{i}^{(r)T}{\text{Σ}}^{-1}y_{i}\right)\right],\\ & V_{\beta }={\left({\text{Σ}}_{\beta }^{-1}+\overset{m_{j}}{\underset{i=1}{\sum }}X_{i}^{(r)T}{\text{Σ}}^{-1}X_{i}^{\left(r\right)}\right)}^{-1}. \end{array}$$

Similarly, the full conditional distributions for the other parameters can be derived as follows:

(A.4) $$\begin{array}{cc} & \pi \left(\gamma \;|\;\cdot \right)\propto \left[\exp\left(\gamma \overset{2}{\underset{j=1}{\sum }}\overset{m_{j}}{\underset{i=1}{\sum }}\left(h_{i}\left(s_{i}\right)\beta_{j}^{T}+g_{i}\left(s_{i}\right)\theta_{i}^{T}\right)\right)\right]\\ & \;\;\;\;\times \left[\overset{n}{\underset{i=1}{\prod }}S_{i}\left(s_{i}\right)\right].\\ & \pi \left(\eta \;|\;\cdot \right)\propto \left[\overset{n}{\underset{i=1}{\prod }}\det{\left(\text{Σ}\right)}^{-(1/2)}\right]\text{f}\\ & \;\;\;\times \exp[-\frac{1}{2}\eta^{\prime }{\text{Σ}}_{\eta }^{-1}\eta -\frac{1}{2}\overset{2}{\underset{j=1}{\sum }}\overset{m_{j}}{\underset{i=1}{\sum }}(y_{i}-X_{i}^{\left(r\right)}\beta_{j}^{T}\\ & \;\;\;\;\;{\times -X_{i}^{\left(m\right)}\theta_{i}^{T})}^{T}{\text{Σ}}^{-1}\left(y_{i}-X_{i}^{\left(r\right)}\beta_{j}^{T}-X_{i}^{\left(m\right)}\theta_{i}^{T}\right)].\\ & \pi \left(\delta \;|\;\cdot \right)\propto \left[\overset{n}{\underset{i=1}{\prod }}\det{\left(\text{Σ}\right)}^{-(1/2)}\right]\\ & \;\;\;\times \exp[-\frac{1}{2}\delta^{\prime }{\text{Σ}}_{\delta }^{-1}\delta -\frac{1}{2}\overset{2}{\underset{j=1}{\sum }}\overset{m_{j}}{\underset{i=1}{\sum }}(y_{i}-X_{i}^{\left(r\right)}\beta_{j}^{T}\\ & \;\;\;\;\;{\times -X_{i}^{\left(m\right)}\theta_{i}^{T})}^{T}{\text{Σ}}^{-1}\left(y_{i}-X_{i}^{\left(r\right)}\beta_{j}^{T}-X_{i}^{\left(m\right)}\theta_{i}^{T}\right)].\\ & \pi \left(\sigma^{2}\;|\;\cdot \right)\propto \left[\overset{n}{\underset{i=1}{\prod }}\det{\left(\text{Σ}+\sigma^{2}X_{i}^{\left(m\right)}X_{i}^{(m)T}\right)}^{-(1/2)}\right]\\ & \;\;\;\;\;\times {\left(\sigma^{2}\right)}^{-\alpha_{1}-1}\exp\left(-\frac{\alpha_{2}}{\sigma^{2}}\right)\\ & \;\;\;\;\times \exp[-\frac{1}{2}\overset{2}{\underset{j=1}{\sum }}\overset{m_{j}}{\underset{i=1}{\sum }}{\left(y_{i}-X_{i}^{\left(r\right)}\beta_{j}^{T}\right)}^{T}\\ & \;\;\;\;\;\;{\times \left(\text{Σ}+\sigma^{2}X_{i}^{\left(m\right)}X_{i}^{(m)T}\right)}^{-1}\left(y_{i}-X_{i}^{\left(r\right)}\beta_{j}^{T}\right)].\\ & \pi \left(\lambda_{0k}\;|\;\cdot \right)\propto \text{Gamma}\left[d_{k}+1,\phi_{\lambda_{0k}}^{-1}\right]\pi \left(\lambda_{0k}\right)\\ & \;\;\;\;=\text{Gamma}\left(a+d_{k}+1,{\left[\phi_{\lambda_{0k}}+\frac{1}{b}\right]}^{-1}\right), \end{array}$$

where *d*_*k*_ is the number of events in the interval (*t*_*k*−1_^*λ*^, *t*_*k*_^*λ*^] and

(A.5) $$\begin{array}{cc} & \phi_{\lambda_{0k}}=\sum_{i:s_{i}\geq t_{k}^{\lambda }}\overset{t_{k}^{\lambda }}{\underset{t_{k-1}^{\lambda }}{\int }}\exp\;\left[\gamma \left(h\left(t\right)\beta_{j}^{T}+g\left(t\right)\theta_{i}^{T}\right)\right]dt\\ & \;\;+\sum_{i:t_{k-1}^{\lambda }<s_{i}\leq t_{k}^{\lambda }}\overset{s_{i}}{\underset{t_{k-1}^{\lambda }}{\int }}\exp\;\left[\gamma \left(h\left(t\right)\beta_{j}^{T}+g\left(t\right)\theta_{i}^{T}\right)\right]dt. \end{array}$$
